# Comparison of Outcomes Derived from the ActiGraph GT3X+ and the Axivity AX3 Accelerometer to Objectively Measure 24-Hour Movement Behaviors in Adults: A Cross-Sectional Study

**DOI:** 10.3390/ijerph19010271

**Published:** 2021-12-27

**Authors:** Marieke De Craemer, Vera Verbestel

**Affiliations:** 1 Department of Rehabilitation Sciences, Faculty of Medicine and Health Sciences, Ghent University, Corneel Heymanslaan 10, 9000 Ghent, Belgium; Vera.Verbestel@UGent.be; 2 Research Foundation Flanders, Egmontstraat 5, 1000 Brussels, Belgium

**Keywords:** physical activity, sedentary behavior, sleep, accelerometer, adult

## Abstract

The aim of this cross-sectional study is to investigate convergent validity of outcomes derived from the ActiGraph GT3X and Axivity accelerometer and to investigate feasibility and acceptability of using outcomes derived from these devices to measure 24-h movement behaviors (i.e., sleep, sedentary behavior, and physical activity) in adults during free-living conditions. Twenty-four adults (33.3% male; 39.79 ± 13.09 years old) simultaneously wore a hip-mounted ActiGraph and thigh-mounted Axivity during 48 consecutive hours. The ActiGraph was switched from hip to wrist during the night. To assess convergent validity between the two devices, Paired sample *t*-tests, linear regressions, Bland–Altman plots and *κ* statistic were conducted. Feasibility and acceptability of the devices was self-reported on a five-point scale. Daily time spent in 24-h movement behaviors differed between both devices. Except for sleep, the mean differences in daily time spent in the behaviors were clinically relevant and the limits of agreement were wide. For all 24-h movement behaviors, except for sleep and vigorous physical activity, proportional errors were observed. κ statistic showed substantial agreement for total physical activity and outstanding agreement for sedentary behavior and sleep. Adults perceived the Axivity as more practical than the ActiGraph to wear it for more than two days whereas the feasibility to wear it for two days was comparable. Depending on the measure, the observed biases were clinically relevant, proportional to the size of the measure and/or erratically variable. When taking into account the data processing approaches applied in this study, the ActiGraph and the Axivity cannot be used interchangeably to measure 24-h movement behaviors since the bias between both devices is clinically relevant and unpredictable.

## 1. Introduction

Recently, a shift in research occurred going from studying movement behaviors in isolation (e.g., physical activity (PA)) towards an integrated approach in which all behaviors conducted within a 24-h time span are distributed across a movement continuum going from no movement (sleep, sedentary behavior (SB)) to movement (categorized from low movement (light-intensity PA or LIPA) towards moderate PA (MPA) and vigorous PA (VPA)). These behaviors interact, which means that time spent on one or more of these behaviors (e.g., PA) has a consequence on the time that can be spend on the other behaviors (i.e., SB and sleep) [1].

The Canadian Society for Exercise Physiology already acknowledged the importance of all movement behaviors in a 24-h period for overall health by incorporating these behaviors in their current guidelines for adults [2]. In addition, very recent studies looked at the combination of PA, SB, and sleep and associations with, e.g., mortality [3], adiposity, cardiometabolic biomarkers (e.g., glucose, insulin control, and lipids) [4], and mental health [5]. In addition, a recent systematic review by Janssen et al. (2020) found that reallocating time from movement behaviors into moderate-to-vigorous PA (MVPA) was associated with favorable changes to several health outcomes [6]. The studies included in this review primarily assessed sleep using self-reports, in combination with objective methods to quantify PA and SB. However, it might be interesting to study the distribution of time spent in PA, SB, and sleep using devices to capture the full 24-h time span in an objective manner.

Accelerometers are widely used to measure PA and SB [7]. Although polysomnography is the most accepted method to objectively measure sleep [8], ActiGraphy has been developed as an alternative method to quantify sleep, is often validated against polysomnography and showed high rates of agreement [7,9]. The relevance of using accelerometers to measure the full 24-h cycle, including PA, SB, and sleep, becomes more apparent [8]. The study of Rosenberger et al. (2016) performed important ground-work by investigating the accuracy of nine different, small wearable wrist-worn devices (e.g., ActiGraph GT3X+, Fitbit, Jawbone, ActivPAL) to measure 24-h movement behaviors (24-HMBs). The closest measurement for sleep was found for the ActiGraph GT3X+, which was not found for PA. This might be due to the fact that the ActiGraph was worn at the wrist, since the device showed to be the best field-based criterion measure of free-living PA and SB using hip placement [10]. For that reason, it is important to switch the device between hip placement (during the day) and wrist placement (during the night) which in turn induces some impracticalities.

Next to accuracy, the acceptability and feasibility of using devices to objectively capture the full 24-h composition is important. One of the emerging devices to objectively measure 24-h movement behaviors (24-HMBs) that was not included in the study of Rosenberger et al. (2016), is the Axivity accelerometer (www.axivity.com, accessed 15 October 2021). The Axivity provides raw acceleration data and open-source software which offers the opportunity to use it on a larger scale without additional costs than purchase. Since the device can be worn at different body locations (e.g., wrist, thigh, and lower back), it is possible to wear the device for a long period of time without having to switch between locations. Therefore, the aim of this cross-sectional study was (1) to investigate the convergent validity of outcomes derived from the ActiGraph and the Axivity and (2) to investigate the feasibility and acceptability of using these devices to measure 24-HMBs in adults.

## 2. Methods

A cross-sectional study was conducted between March and April 2021. Convenience and snowball sampling were used to recruit adults (18–65 years of age) in Flanders, Belgium. Participants were contacted by telephone, after which a home visit was performed by the researcher to explain the aim of the study and to mount the devices. Adults provided an active informed consent prior to data collection. All participants had to wear two accelerometers (i.e., ActiGraph GT3X+ and Axivity AX3) during 48 consecutive hours (i.e., during waking hours and during sleep). Both devices were collected after the measurement period had ended. This study was approved by the Ethics Committee of the Ghent University Hospital (BC-08863).

The first device was the ActiGraph GT3X+ triaxial accelerometer, which is lightweight (19 g) and small (46 × 33 × 15 mm). The accelerometer was initialized to start measuring simultaneously with the Axivity using ActiLife v6.13.4 software. Since PA and SB are more accurately measured with hip placement [7], participants had to wear the ActiGraph at the right hip above the iliac crest using an elastic waist band during the day. The ActiGraph was switched from hip to wrist during the night using wrist bands since sleep is more accurately measured during wrist placement compared to hip placement [7]. Participants were instructed to only remove the device for water-based activities such as showering or swimming.

The Axivity (AX3; Axivity Ltd, Newcastle Upon Tyne, United Kingdom.) is a data logger including a MEMS 3-axis accelerometer which enables the device to collect movement data. It is a small (23 × 32.5 × 7.6 mm), lightweight (11 g) and unobtrusive device. Before the start of data collection, the Axivity was synchronized and initialized to start measuring simultaneously with the ActiGraph using the Open Movement software (OMGui, version 1.0.0.43; Newcastle University, UK). First, the Axivity was made waterproof by covering the device with a thin finger cot and a transparent 3M Tegaderm tape. Then, it was mounted on the participant’s right anterior thigh midway between the hip and knee (i.e., the same side of the body as for the ActiGraph), using a second transparent and hypoallergenic 3M Tegaderm tape (10 × 10 cm).

Participants reported year of birth, sex, height, and weight. Height and weight were used to calculate participants’ Body Mass Index (BMI; weight/height in m²). Participants rated the acceptability of the devices using a five-point scale (“very pleasant”; “pleasant”; “neutral”; “unpleasant”; and “very unpleasant”) on the following questions: “How did you experience wearing the Axivity accelerometer on your upper thigh?”, “How did you experience wearing the ActiGraph accelerometer at your hip during the day?”, and “How did you experience wearing the ActiGraph accelerometer at your wrist during the night?”. In addition, participants rated the feasibility to wear the devices using a three-point scale (“yes”, feasible for 48 h or even longer”; “yes, feasible for a couple of hours”; “no, not feasible”) on the following questions: “How did you perceive the feasibility of wearing the Axivity accelerometer at your upper thigh?”, “How did you perceive the feasibility of wearing the ActiGraph accelerometer at your hip during the day?”, and “How did you perceive the feasibility of wearing the ActiGraph accelerometer at your wrist during the night?”.

To be included in the analyses, concurrent data of both devices had to be available for 48 consecutive hours. ActiGraph data were downloaded in 60-s epochs using ActiLife software. Freedson cut-points [11] were used to categorized counts as either SB (<100 counts/min), LIPA (100–1951 counts/min), MPA (1952–5724 counts/min) or VPA (>5724 counts/min). By means of “0” (=no) or “1” (=yes), it was reflected for each minute whether the participant engaged in SB, LIPA, MPA, or VPA. This minute-by-minute categorization was exported into an Excel-file. In addition, the sleep algorithm available through the ActiLife software was used to determine periods of sleep. For every minute, it was reflected whether the participant was asleep (=1) or awake (=0). These sleep data were combined with the activity data to determine periods of PA, SB or sleep. This resulted in 2880 min of categorized data in which the sum of LIPA, MPA, and VPA reflected total PA, and the sum of MPA and VPA reflected MVPA. The sum of all minutes for each category (i.e., PA, SB and sleep) was made to reflect the total amount of daily minutes spent in PA, SB or sleep across a full 24-h day when using an ActiGraph.

Axivity data were downloaded and processed in 60-s epochs using the default cut-points available in the Open Movement software (OMGui). The result was an Excel-file in which every minute was coded as either SB or PA. The same 48-h block of data from the ActiGraph was selected from the Axivity. There were four different columns in the Excel-file, one column for SB, one column for LIPA, one column for MPA and one column for VPA. By means of “0” (=no) or “1” (=yes), it was reflected for each minute whether the participant engaged in SB, LIPA, MPA, or VPA. In addition, a second Excel-file was obtained through the Open Movement software in which all periods of participants’ sleep were reflected minute-by-minute, and this was aggregated with the periods of SB and PA in the Excel-file. In the end, the sum of all minutes was made to reflect the total amount of daily minutes spent in PA, SB, or sleep across a full 24-h day when using an Axivity.

Analyses were performed using SPSS for Windows version 27.0. Descriptive statistics were used to describe sample characteristics, feasibility, and acceptability of both devices, and to express the percentage of time spent in PA, SB and sleep as measured by the ActiGraph and Axivity. Paired sample *t*-tests were performed to analyze the difference in mean minutes of total PA, LIPA, MPA, VPA, MVPA, SB, and sleep across 24 h measured by the ActiGraph and the Axivity. A *p*-value of <0.05 was seen as statistically significant. Bland–Altman plots were used to investigate the difference in group mean minutes per day between the devices. Limits of agreement were set at ± 1.96 standard deviations of the mean differences [12]. As a supplement to the Bland–Altman plots, linear regression analyses were conducted to investigate if the bias was constant across the range of measurements [12]. Linear regression analyses were conducted for each measure separately with the difference between the two devices as the dependent variable and the mean of the two devices as an independent predictor. Finally, κ statistic was used as an indication of agreement between the binary coded minute-by-minute PA versus non-PA (including LIPA, MPA, VPA, and MVPA), SB versus non-SB and sleep versus non-sleep. Agreement was categorized as moderate (κ = 0.40–0.59), substantial (κ = 0.60–0.79), or outstanding (κ ≥ 0.80) [13]. Effect sizes were calculated by using the following formula: θ = (μ_1_ − μ_2_)/σ with θ as effect size, μ_1_ as the mean of device 1, μ_2_ as the mean of device 2, and σ as standard deviation.

## 3. Results

In total, 24 participants (33.3% male; 39.79 ± 13.09 years old (range: 19–62)) consented to participate in the study. All participants (100%) provided valid data for 48 h which corresponds to 69,120 valid minutes of categorized data. Of those 24, 19 participants (79.2%) had a normal weight, four (16.7%) had overweight and one (4.2%) did not report height and weight.

**Figure 1:**
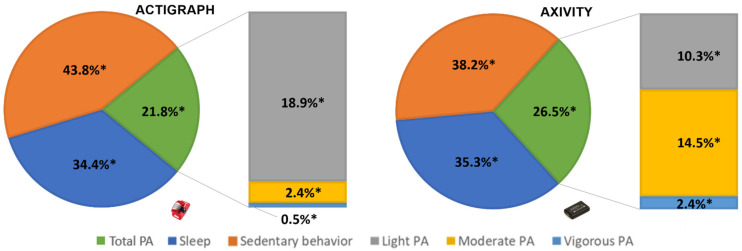
Distribution of time in 24-h movement behaviors as measured with the ActiGraph and the Axivity. Abbreviation: PA = physical activity * Significant difference between ActiGraph and Axivity.

Table 1 and Figure 1 show the percentage and minutes of time spent in objectively measured PA (total PA, LIPA, MPA, VPA, and MVPA), SB and sleep measured by the ActiGraph and the Axivity. Paired sample *t*-tests showed a significant difference in daily time spent in SB (t = 8.42, *p* < 0.001), sleep (t = −3.42, *p* = 0.001), LIPA (t = 12.57, *p* < 0.001), MPA (t = −11.39, *p* < 0.001), VPA (t = −4.30, *p* < 0.001), MVPA (t = −13.79, *p* < 0.001), and total PA (t = −6.89, *p* < 0.001) between both devices (Table 1). Linear regression analyses demonstrated a significant negative trend in bias for SB (F = 7.78, *p* = 0.011), MPA (F = 56.61, *p* > 0.001), MVPA (F = 44.56, *p* > 0.001) and total PA (F = 8.63, *p* = 0.008). A significant positive trend in bias was found for LIPA (F = 29.34, *p* < 0.001). No trend in bias was observed for sleep (F = 3.84, *p* = 0.063) and VPA (F = 4.24, *p* = 0.052). The regression equations can be found in Table 1. The mean differences, limits of agreement and trends in biases are graphically presented in the Bland–Altman plots (Supplementary Materials). When comparing minute-by-minute data, κ statistic showed substantial agreement for total PA (κ = 0.77, *p* < 0.001). Outstanding agreement was found for SB (κ = 0.81; *p* < 0.001) and sleep (κ = 0.96; *p* < 0.001). No agreement was found for LIPA, MPA, VPA, and MVPA (all κ < 0.30). The adults’ perceived acceptability and feasibility of the devices can be found in Table 2.

## 4. Discussion

The main aim of this study was to investigate the comparability of outcomes related to 24-HMBs between the ActiGraph and Axivity in adults. The major finding was that there were considerable disagreements between outcomes derived from these devices to measure 24-HMBs in adults. This results in considerable differences in adults’ 24-HMBs and impacts adherence rates with 24-h movement behavior guidelines depending on the device that is used.

At group level, the Axivity overestimated daily time spent in sleep and high(er) intensity related PA (MPA, VPA, and MVPA) relative to the ActiGraph. In contrast, the Axivity underestimated daily time spent in sleep and SB. Although minute-by-minute agreement between the ActiGraph and the Axivity was promising for sleep and SB, a lack of data convergence was observed between all 24-HMBs. This lack of convergence is due to several types of data anomalies that were present among most outcomes. First, the biases were related to the size of the measure in all outcomes except for sleep and VPA. The results also demonstrated that all outcome measures, except for SB, were erratically variable. This means that more than 6% of the data points lied outside the limits of agreements which suggests that the disagreements between both methods cannot be easily corrected. As recommended by Riffenburgh et al. (2020), a larger sample size (i.e., more than 100 data points) is needed to test the consistency of this data aberration outside the limits of agreement [14]. Last, the biases that were observed were clinically relevant in all outcomes, except for sleep where a small mean difference and narrow limits of agreement were observed. The limits of agreements of all other outcomes were wide and the differences meaningfully affect the interpretation of results. For MVPA for example, the limits of agreement range over an interval of 68 min/day. Given that MVPA generally accounts for a small percentage across the day and the fact that 150 min of MVPA is recommended for adults on a weekly basis to obtain health benefits [2], a difference that corresponds to more than 1 h per day is epidemiologically and clinically unacceptable.

To our knowledge, this is the first study that investigated the convergence of 24-HMBs between the ActiGraph and Axivity in adults in free-living conditions. Studies comparing 24-HMBs in isolation were also considered but we did not find studies whose results were directly comparable with this study. The comparability of studies reporting on the convergence between ActiGraph and Axivity to measure PA and/or SB was hampered by: (1) the variability in the location where one or both devices were worn (e.g., hip versus wrist) [15], (2) behavioral conceptualization (e.g., steps versus intensity) [16], (3) the setting (e.g., field-based versus laboratory) [17], and (4) the use of different data processing methods (e.g., GGIR versus OMGui) [18]. Although we found no studies of which the results were generalizable to this study, our results are consistent with the major finding of Rosenberger and colleagues’ (2016) review that several wearable devices are not able to accurately measure MVPA (with the ActiGraph used as criterion measure). This review compared the accuracy of nine devices to measure 24-HMBs but the Axivity was not included. Accurately measuring MVPA is very important as it is the outcome measure for which the most evidence is available in relation to health outcomes and together with SB, it is detrimental for correctly classifying LIPA.

The lack of convergence between 24-HMBs as measured by the ActiGraph and the Axivity might be explained by several factors. First, the ActiGraph is a common field-based measure in epidemiological studies but it is not a golden standard to measure 24-HMBs in adults. Thus, using the ActiGraph as the reference method might have introduced significant errors in the comparison. The 24-HMBs in this study were expressed in daily time in sleep, SB, LIPA, and MVPA as defined by the Freedson cut-points [11]. Exploring the convergence of intensity related activities, including the use of these cut-points, is highly relevant as they are commonly used in epidemiological studies. However, the use of cut-points to determine SB and intensity-related PA adds an additional source of error in the classification of the behaviors. For example, using hip-mounted ActiGraph accelerometers has the limitation that it classifies standing (relatively) still as SB instead of LIPA. This misclassification implies that an overestimation of SB and an underestimation of LIPA might have hampered the agreement between the outcomes. Last, as it is not known how the software determines intensity related PA from the Axivity data, the observed differences may be due to the definitions and data processing methods used.

A second aim of the study was to investigate adults’ perception about the acceptability and feasibility of both devices. The main finding was that adults perceived the Axivity as more practical to wear for more than two days than the ActiGraph whereas the feasibility to wear it for two days was comparable. This is not surprising as the ActiGraph needs to be switched from the hip to the wrist in the evening and to be re-attached to the hip in the morning which adds additional burden to the participant. Based on feasibility and acceptability, the Axivity appears to be a practical alternative for the ActiGraph in large scale studies. The use of the Axivity might increase wear time compliance [19] and improved data quality but further research is needed to clarify its validity to measure 24-HMBs in adults.

Some limitations of the present study need to be acknowledged. This study was limited by the use of a relatively small convenience sample which might have resulted in a selection bias. Given the unpredictable data aberration observed, this study should be replicated in a larger sample [14] that should be recruited by using probability sampling. In addition, it should be acknowledged that we did not compare raw accelerations from both measurement devices, but we compared outcomes based on different processing decisions. This implies that different outcomes might be retrieved if other data processing decisions are applied. It is important to be aware of this limitation since both devices cannot be used interchangeably based on the processing decisions that have been made in the current study. Furthermore, the ActiGraph is a common field-based measure of 24-HMBs in adults but does not represent a gold standard. This implies that the results of this study do not allow to claim the ActiGraph to be superior over the Axivity to measure 24-HMBs in adults.

## 5. Conclusions

The Axivity is perceived a practical and feasible device for adults, but considerable disagreements in 24-HMBs were found with the ActiGraph. Depending on the measure, the observed biases were clinically relevant, proportional to the size of the measure and/or erratically variable. Based on the data processing approaches applied in this study, the ActiGraph and the Axivity cannot be used interchangeably to measure 24-HMBs as the bias between the devices is clinically relevant and unpredictable.

## Figures and Tables

**Table 1:** Bland–Altman analyses for agreement between the ActiGraph and Axivity defined mean time in 24-h movement behaviors and linear regression analyses to investigate trends in bias between the devices in the total sample (n = 24).

24-HMBs	ActiGraph			Axivity				Comparison between Both Devices		
								Agreement		Trends in Bias
	% of 24 h	Median (Min/Day)	Min–Max (Min/Day)	% of 24 h	Median (Min/Day)	Min–Max (Min/Day)	Effect Size	Mean Difference	95% Limits of Agreement	Constant (B)
Total PA	21.77	282.00	136–584	24.96	317.50	146–686	0.33	−45.92 *	−66.33, −25.50	17.39 (−0.19) ⱽ
LIPA	18.87	240.00	118–540	10.14	132.50	55–352	1.44	125.71 *	99.77, 151.65	12.34 (0.54) ⱽ
MPA	2.38	23.00	4–138	13.42	147.00	68–414	2.45	−159.04 *	−193.49, −124.60	−27.86 (−1.15) ⱽ
VPA	0.52	0.00	0–137	1.39	3.50	0–156	0.39	−12.58 *	−21.95, −3.22	−8.48 (−0.29)
MVPA	2.90	26.50	4–147	14.81	181.00	68–416	2.42	−171.63 *	−205.63, −137.62	−44.96 (−0.99) ⱽ
SB	43.81	636.50	410–812	39.65	612.00	299–805	0.45	59.88 *	37.52, 82.23	182.92 (−0.21) ⱽ
Sleep	34.42	499.00	424–585	35.39	494.50	442–619	0.29	−13.96 *	−21.64, −6.27	60.09 (−0.15)

Abbreviations: 24-HMBs = 24-h movement behaviors, PA = physical activity; LIPA = light intensity PA, MPA = moderate intensity PA, VPA = vigorous intensity PA, MVPA = moderate-to-vigorous PA, SB = sedentary behavior, SD = standard deviation, B = unstandardized beta. * Significant difference between ActiGraph and Axivity (*p* < 0.01). ⱽ Significant linear regression model (dependent variable: difference between the two devices; independent predictor: mean of the two devices; *p* < 0.05) indicating a trend in bias between the devices.

**Table 2:** Descriptive statistics of adults’ perceived acceptability and feasibility to wear the ActiGraph (on the hip during the day and at the wrist during the night) and the Axivity in the total sample (n = 24).

	ActiGraph (Hip–Waking Hours)	ActiGraph (Wrist–Sleep)	Axivity
Acceptability to wear the device			
Pleasant to very pleasant	25.00%	20.80%	62.50%
Neutral	50.00%	66.70%	33.30%
Unpleasant to very unpleasant	20.80%	12.50%	0%
No response	4.20%	0%	4.20%
Feasibility to wear the device			
For ≥48 h	87.50%	79.20%	95.80%
For a couple of hours	12.50%	20.80%	0%
Not feasible	0%	0%	0%
No response	0%	0%	4.20%

## Data Availability

Data sharing is applicable upon request.

## Supplementary Materials

The following are available online at https://www.mdpi.com/article/10.3390/ijerph19010271/s1, Figure S1: Sleep, Figure S2: Sedentary behaviour, Figure S3: Light intensity physical activity (LIPA), Figure S4: Moderate intensity physical activity (MPA), Figure S5: Vigorous intensity physical activity (VPA), Figure S6: Moderate-to-vigorous intensity physical activity (MVPA), Figure S7: Total physical activity (PA).
