# Supplementary material for: Comparison of Outcomes Derived from the ActiGraph GT3X+ and the Axivity AX3 Accelerometer to Objectively Measure 24-Hour Movement Behaviors in Adults: A Cross-Sectional Study
Source: Int J Environ Res Public Health. 2021 Dec 27;19(1):271. doi: 10.3390/ijerph19010271 (PMC8750776; doi:10.3390/ijerph19010271)
Supplement: Supplementary file 1 [file ijerph-19-00271-s001.zip › ijerph-1456155-supplementary.pdf]

Supplmentary Materials

Bland-Altman plots for agreement between ActiGraph and Axivity defined mean time spent in sleep (Figure S1), sedentary behavior (Figure S2), light intensity physical activity (PA) (Figure S3), moderate intensity PA (Figure S4), vigorous intensity PA (Figure S5), moderate-to-vigorous intensity PA (Figure S6), and total PA (Figure S7). The *red* line denotes the group mean difference between ActiGraph and Axivity, the *green* lines denote 95% limits of agreement (mean difference ± 1.96 standard deviation) and the *black* lines denote the linear regression line.

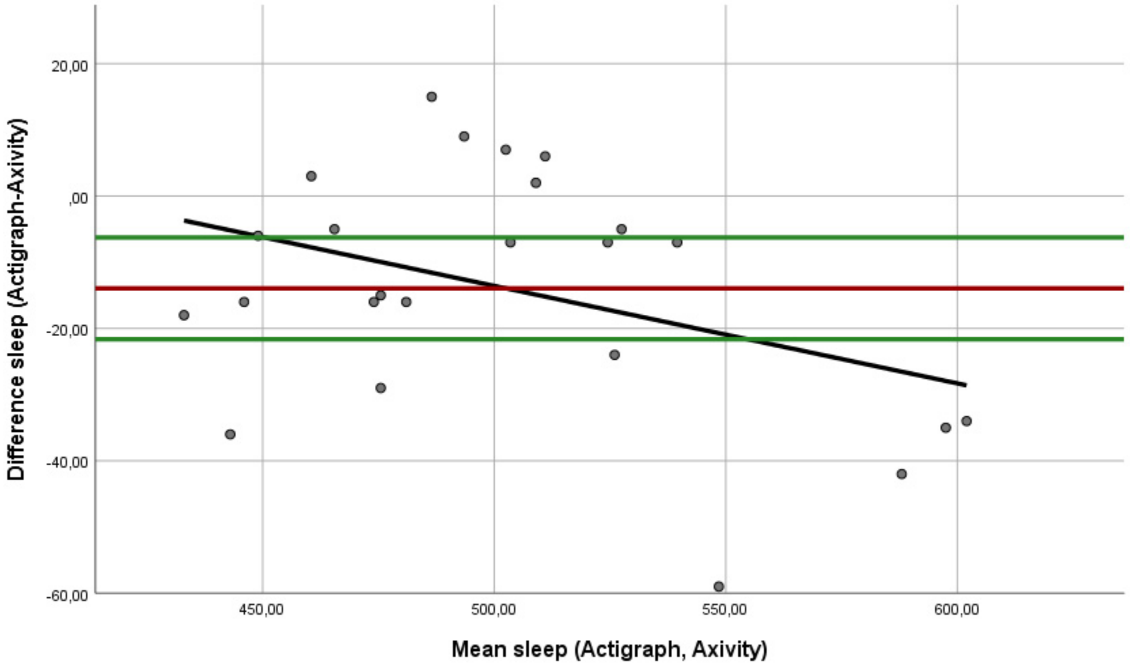

Figure S1. Sleep.

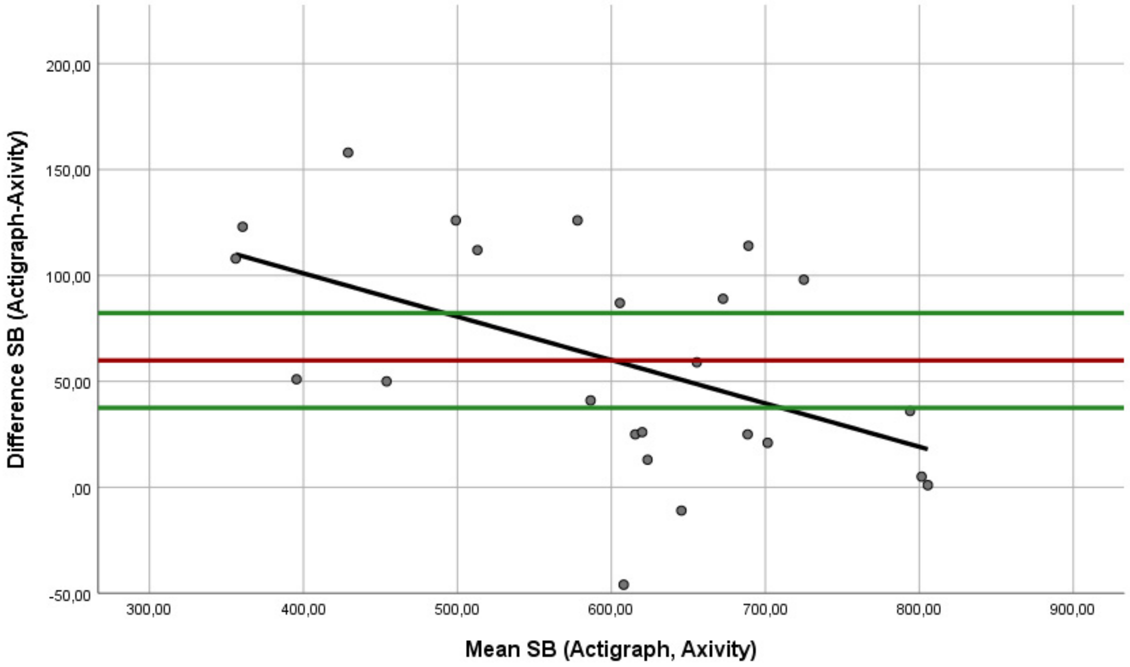

Figure S2. Sedentary behaviour.

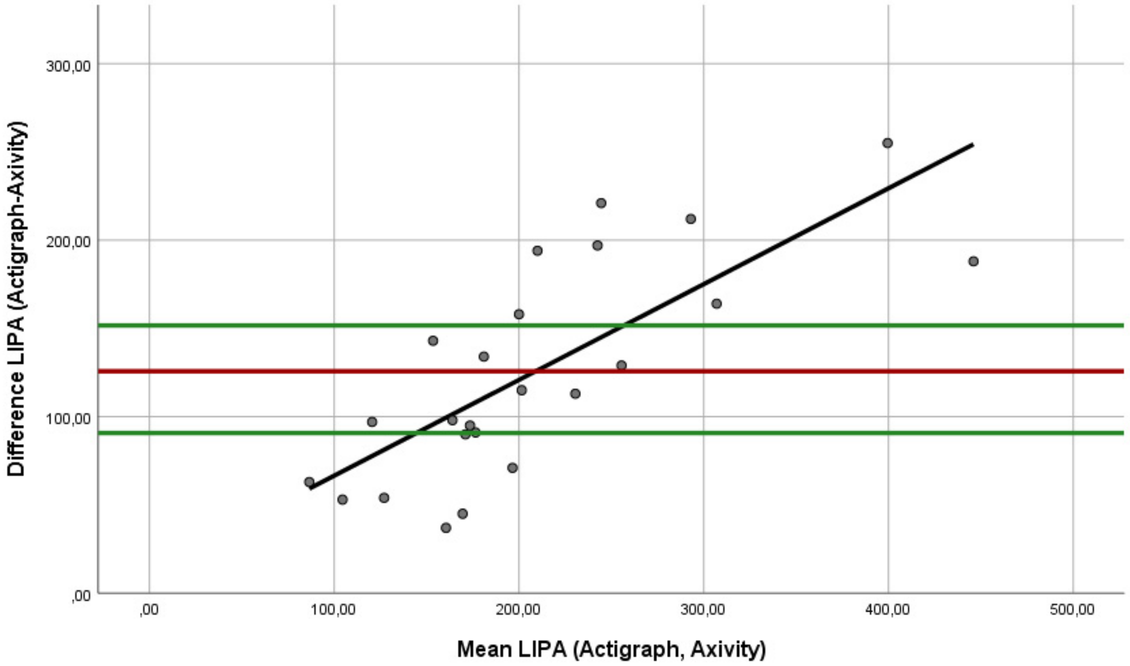

Figure S3. Light intensity physical activity (LIPA).

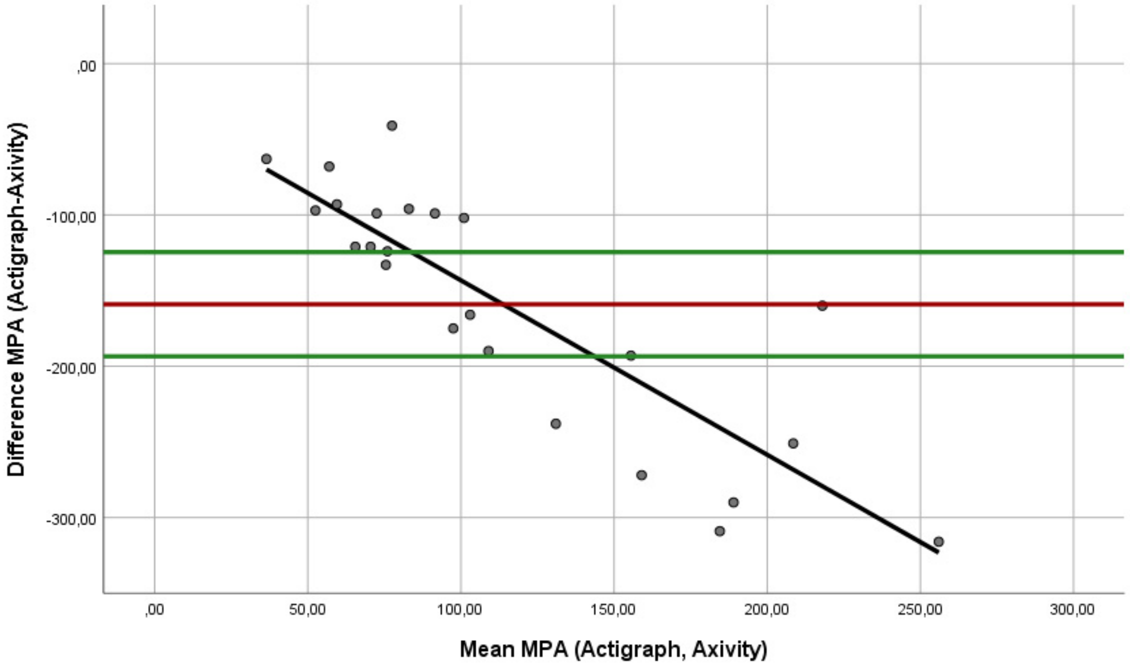

Figure S4. Moderate intensity physical activity (MPA).

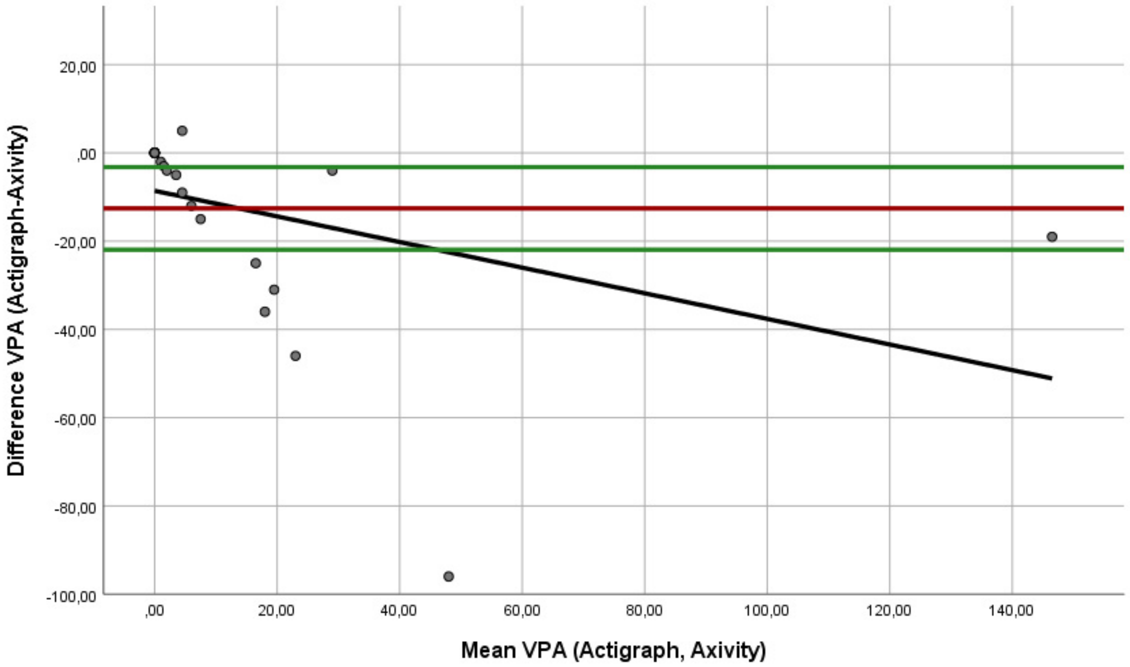

Figure S5. Vigorous intensity physical activity (VPA).

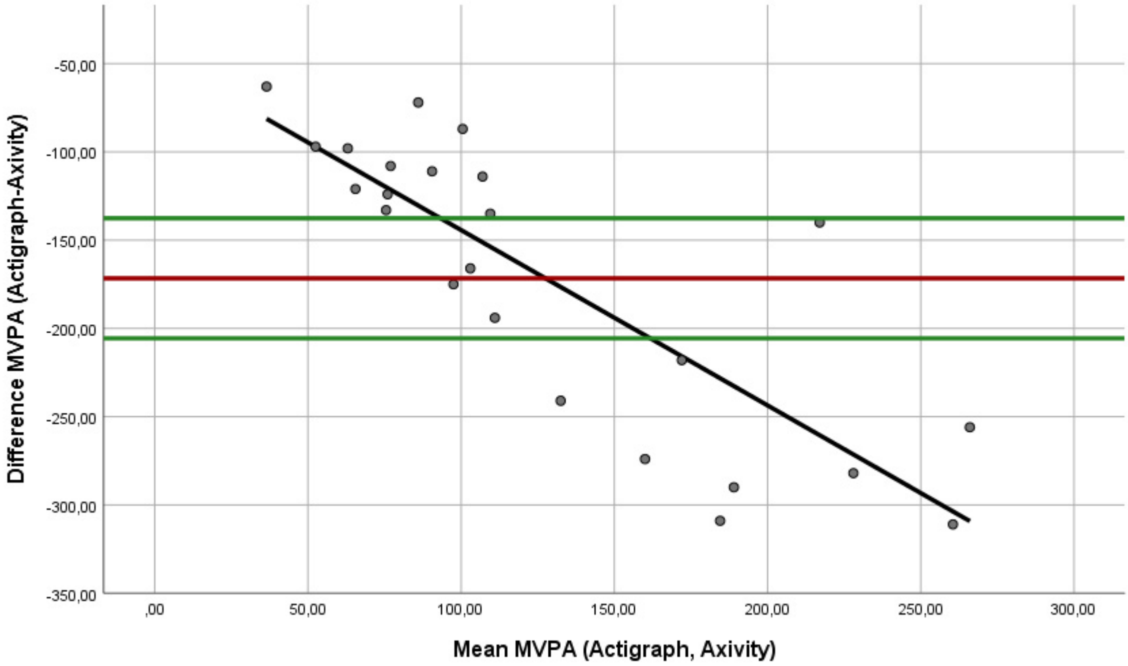

Figure S6. Moderate-to-vigorous intensity physical activity (MVPA).

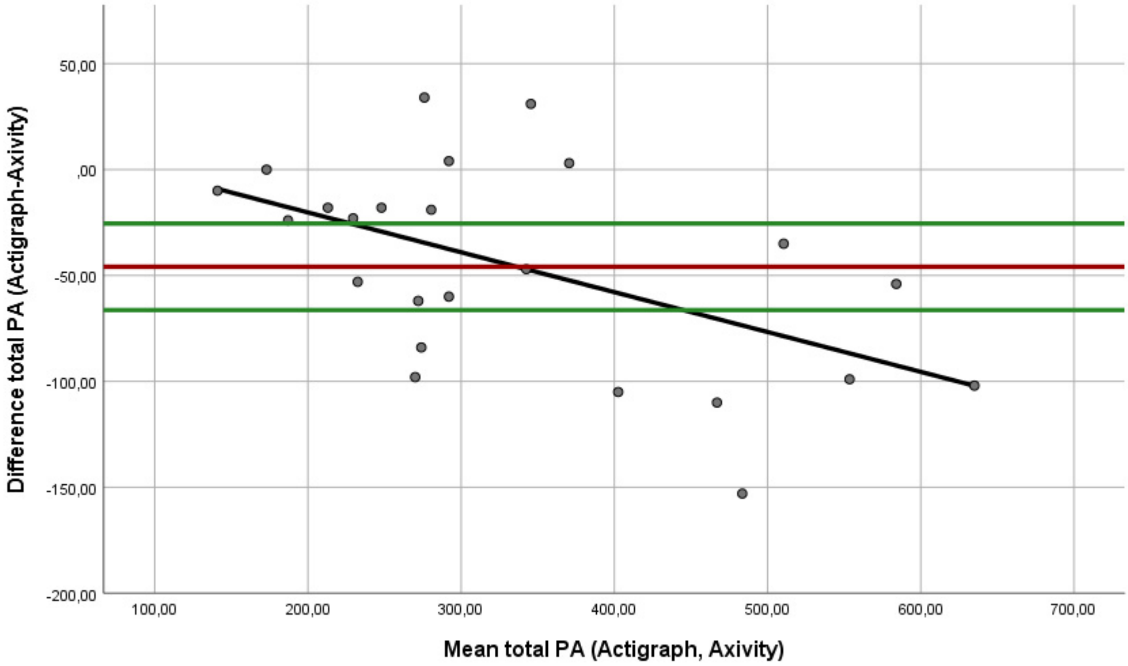

Figure S7. Total physical activity (PA).
